# A New Agricultural Book

**Published:** 1853-07

**Authors:** 


### *A New Agricultural Book. Scientific Agriculture, &c.* By M. M. Rogers, M. D.

This is a book of 300 pages 12mo, with numerous fine illustrations, and neatly printed and bound. Erastus Darrow, Rochester, N. Y., is the publisher, and now issuing the second edition. It is divided into five parts. Part I. contains a well written introduction on the general subjects, a treatise on the elements of chemistry, the proportions of all organic and inorganic bodies necessary to be known to the agriculturist. Part II. contains a clear and concise description of all the rocks and geological phenomena connected with the subject. Part III. treats of the elements of botany, including vegetable physiology, geographical distribution and diseases of plants, &c. Part IV. contains all necessary information on the subject of climates, storms, and other essential phenomena, and also the productions of different latitudes and altitudes. Part V. consists of the application of the foregoing scientific principles to practical agriculture. The subjects of soils, manures, analysis of different grains and soils, rotation of crops, manufacture of butter and cheese, mechanical philosophy, and a variety of other useful topics are clearly and systematically discussed. A copious glossary is appended. We believe this book contains more elementary science than any other of its compass. Every topic treated upon is made clear, and its practical bearing evident; the book can be readily comprehended by any man who can read, and still it is not so simplified as to be destitute of a scientific and erudite character; it is founded upon modern science and undoubted authorities, and ought to, as it doubtless will become a standard work. We understand it has an extensive sale for district school and agricultural libraries. Dr. Rogers is known as the author of a series of letters from Europe, on agricultural subjects. His book has received the most flattering notices from the press of nearly ever state in the Union. B.

